# Aniseikonia after reduced-fluence photodynamic therapy in patients with central serous chorioretinopathy

**DOI:** 10.1038/s41598-023-44415-w

**Published:** 2023-10-10

**Authors:** Aya Chubachi-Kamimura, Akiko Miki, Mayuka Hayashida-Hirano, Maya Kishimoto-Kishi, Mina Okuda, Wataru Matsumiya, Hisanori Imai, Sentaro Kusuhara, Makoto Nakamura

**Affiliations:** Department of Surgery, Division of Ophthalmology, Kobe University Graduate School of Medicine, 7-5-2 Kusunoki-cho, Chuo-ku, Kobe, 650-0017 Japan

**Keywords:** Retinal diseases, Vision disorders

## Abstract

This study investigated aniseikonia after reduced-fluence photodynamic therapy (RFPDT) for central serous chorioretinopathy (CSC). We examined 48 eyes of 48 patients (38 men; mean age, 49.2 ± 9.9 years) with CSC resolved after RFPDT. Horizontal and vertical aniseikonia were measured using the New Aniseikonia Test at baseline, 6 months, and 12 months after RFPDT. The maximum absolute value of the horizontal and vertical measurements indicated the aniseikonia score. The aniseikonia score was 2.2 ± 2.3 at 6 months and 2.2 ± 2.0 at 12 months after RFPDT, both of which improved significantly from the baseline score of 4.1 ± 2.9 (*P* < 0.05 and *P* < 0.01, respectively). The 12-month aniseikonia score significantly correlated with the baseline aniseikonia score (*P* = 0.047), outer nuclear thickness at baseline (*P* = 0.027) and 12 months after RFPDT (*P* = 0.014), baseline SRD area (*P* = 0.005), and ellipsoid zone disruption at 12 months after RFPDT (*P* = 0.021). In multivariate analysis, baseline serous retinal detachment (SRD) area (*P* = 0.034) was significantly associated with aniseikonia score at 12 months after RFPDT. Eyes with a larger SRD area might have higher aniseikonia scores even after SRD resolution following RFPDT.

## Introduction

Central serous chorioretinopathy (CSC) is characterized by the localized detachment of the neurosensory retina at the macula predominantly in middle-aged men^1,2^. Patients with CSC exhibited the following symptoms: minor vision blurring, central scotoma, metamorphopsia, and micropsia^3^. Various studies have reported that reduced-fluence photodynamic therapy (RFPDT) with various modifications of the standard photodynamic therapy (PDT) is effective for the complete resolution of subretinal detachment (SRD) in patients with either acute or chronic CSC^4–8^. However, in clinical settings, physicians encounter patients who have persistent symptoms such as micropsia despite successful treatment^9,10^.

Aniseikonia is a binocular vision anomaly caused by different image sizes perceived between the two eyes. Aniseikonia is associated with anisometropia^11,12^. However, patients with retinal diseases, including reattached retinal detachment (RRD), CSC, and epiretinal membrane, experience aniseikonia^13–16^. The association between aniseikonia and retinal diseases is attributed to variations in perceptual image size caused by the changes in the spacing between photoreceptors resulting from retinal extension or compression^17^. Benegas et al.^13^ reported a case series of patients with diplopia secondary to aniseikonia associated with macular diseases, including ERM. Diplopia may occur when aniseikonia impairs fusion.

However, no clear evidence points to the clinical parameters associated with aniseikonia in patients with CSC. When investigating factors related to aniseikonia, it may be useful to determine the appropriate treatment timing to avoid aniseikonia that disturbs vision quality. Until date, only a few studies have reported aniseikonia in patients with CSC. Therefore, this study aimed to explore the clinical factors related to aniseikonia after RFPDT in patients with CSC.

## Methods

In this study, all procedures involving human participants were performed according to the ethical standards of the institutional and/or national research committee and the 1964 Declaration of Helsinki and its later amendments or comparable ethical standards. The study protocol was approved by the Institutional Review Board of Kobe University Hospital (No.180268). Informed consent was obtained through opt-out on the website.

We retrospectively reviewed 48 eyes of 48 consecutive patients with CSC resolution after RFPDT who were treated between November 2017 and March 2021 at Kobe University Hospital. CSC was diagnosed according to previous reports^7,8,18–20^. Briefly, CSC was defined as the detachment of the neurosensory retina from the macula caused by fluid leakage from the retinal pigment epithelium. Leakage was detected by fluorescein angiography (FA). The inclusion criteria were as follows: (1) presence of subretinal fluid involving the fovea on optical coherence tomography (OCT) images and (2) having at least 1 year of follow-up after RFPDT. The exclusion criteria were as follows: (1) presence of any other ocular diseases that could affect visual acuity, including tilted disc syndrome, a dome-shaped macula, and glaucoma; (2) previous history of PDT or anti-vascular endothelial growth factor therapy, or laser photocoagulation; (3) anisometropia of > 2.0 diopters; and (4) bilateral disease.

All patients underwent complete ophthalmologic examination, including slit-lamp examination, dilated fundus examination, and best-corrected visual acuity (BCVA) measurement (decimal visual acuity) before and after treatment. Macular 6-line radial scan and 31-line raster scan with enhanced depth imaging were performed using the Spectralis OCT System (Heidelberg Spectralis OCT; Heidelberg Engineering GmbH, Heidelberg, Germany) before and after treatment. Digital FA and indocyanine green angiography (ICGA) were performed using the Spectralis OCT System (Heidelberg Spectralis HRA2; Heidelberg Engineering GmbH). In this study, the aniseikonia score, BCVA, and OCT parameters at baseline, 6 months, and 12 months after RFPDT were recorded.

OCT measurements were evaluated according to our previous report^7,10^. The SRD width was defined as the distance on the bottom side of the subretinal space. The average horizontal and vertical widths were used in the analysis. The SRD area was manually measured on infrared fundus photographs using an OCT device. Two independent raters measured the SRD area. As evaluating the disruption of the ellipsoid zone (EZ) layer at baseline was sometimes difficult because of the SRD or photoreceptor outer segment, it was evaluated 6 and 12 months after RFPDT. The degree of aniseikonia was evaluated using the New Aniseikonia Test (NAT; Handaya, Tokyo, Japan). The NAT dissociates binocular vision using a red–green filter to measure the percentage of aniseikonia. It consists of a book and a pair of red–green glasses. A pair of red and green half-moons, each vertically aligned in diameter, are printed on each page. Using a red–green glass, a half-moon is visible to each eye. The difference between each pair of half-moons varies by an increment of 1%. The half-moons are arranged in a sequence. The patients observe the pages through red–green glasses to ensure that the right eye observes one of the half-moons in each pair and the left eye observes the other half-moon. Patients report the pair when the two half-moons appear to be of the same size. The percentage of aniseikonia is indicated by the actual size difference between the two half-moons in the pair. Aniseikonia was measured vertically and horizontally. The aniseikonia score was defined as the maximum absolute value of the horizontal and vertical measurements. All patients received half-time PDT as previously described^7–10^. Briefly, patients received an infusion of verteporfin (Visudyne; Novartis, Basel, Switzerland) at 6 mg/m^2^ body surface area over 10 min, and laser was administered 15 min after the initiation of infusion. The standard light intensity was 600 mW/cm^2^, and the irradiation time was shortened to 42 s (half-time PDT). The spot size covered the areas with actively leaking spots on FA images and also covered the areas of choroidal vascular hyperpermeability (CVH) when CVH was confirmed on ICGA images.

The decimal visual acuity was converted into logMAR units for statistical analyses. The differences in logMAR BCVA and aniseikonia score at baseline, 6 months, and 12 months after RFPDT were analyzed using the Wilcoxon signed-rank test with Bonferroni correction. Reliability of SRD area between raters was assessed using two-way random intraclass correlation coefficients (ICC). Spearman’s rank correlation test was used to examine the association between the aniseikonia score 12 months after RFPDT and the clinical parameters at baseline or 12 months after RFPDT. A multiple regression analysis was conducted to identify the independent parameters associated with the aniseikonia score. A *P*-value < 0.05 was considered statistically significant. Statistical analyses were conducted using IBM SPSS Statistics for Windows version 24.0 (IBM Corp., Armonk, NY, USA).

## Results

**Figure 1:**
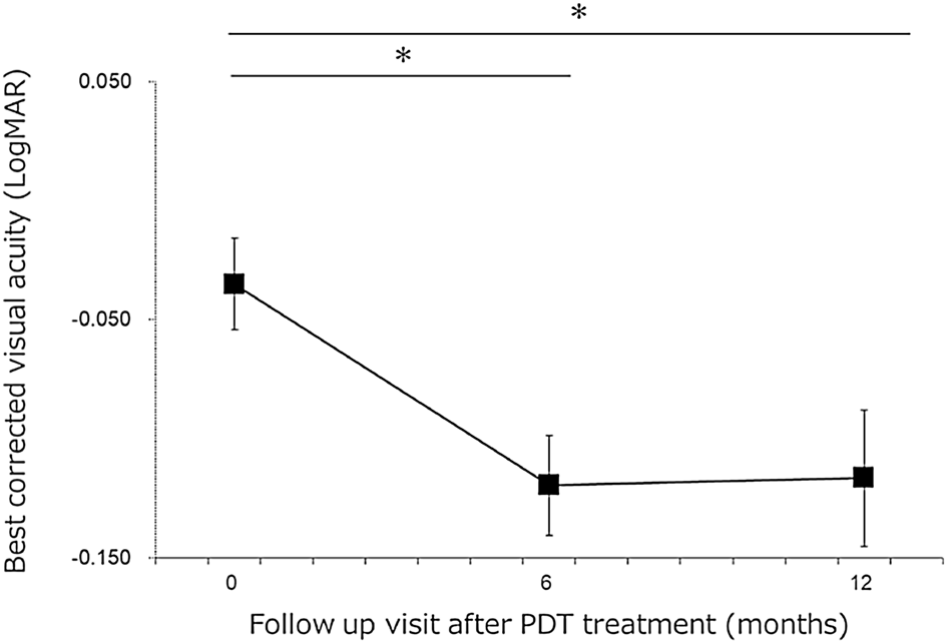
Time course of the best-corrected visual acuity (BCVA) after reduced-fluence photodynamic therapy (RFPDT). The BCVA significantly improved 6 and 12 months after PDT compared with baseline. Error bars mean standard errors. *P < 0.01 (Wilcoxon signed-rank test with Bonferroni correction).

**Figure 2:**
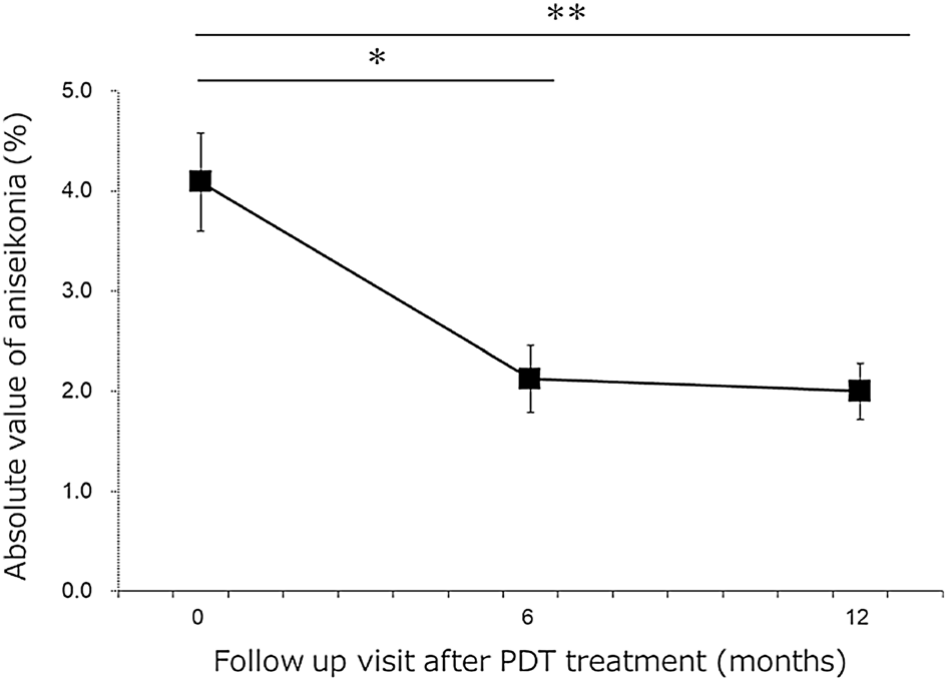
Time course of the aniseikonia score after reduced-fluence photodynamic therapy (RFPDT). Aniseikonia significantly improved 6 and 12 months after PDT compared with baseline. Error bars mean standard errors. *P < 0.05, **P < 0.01 (Wilcoxon signed-rank test with Bonferroni correction).

**Figure 3:**
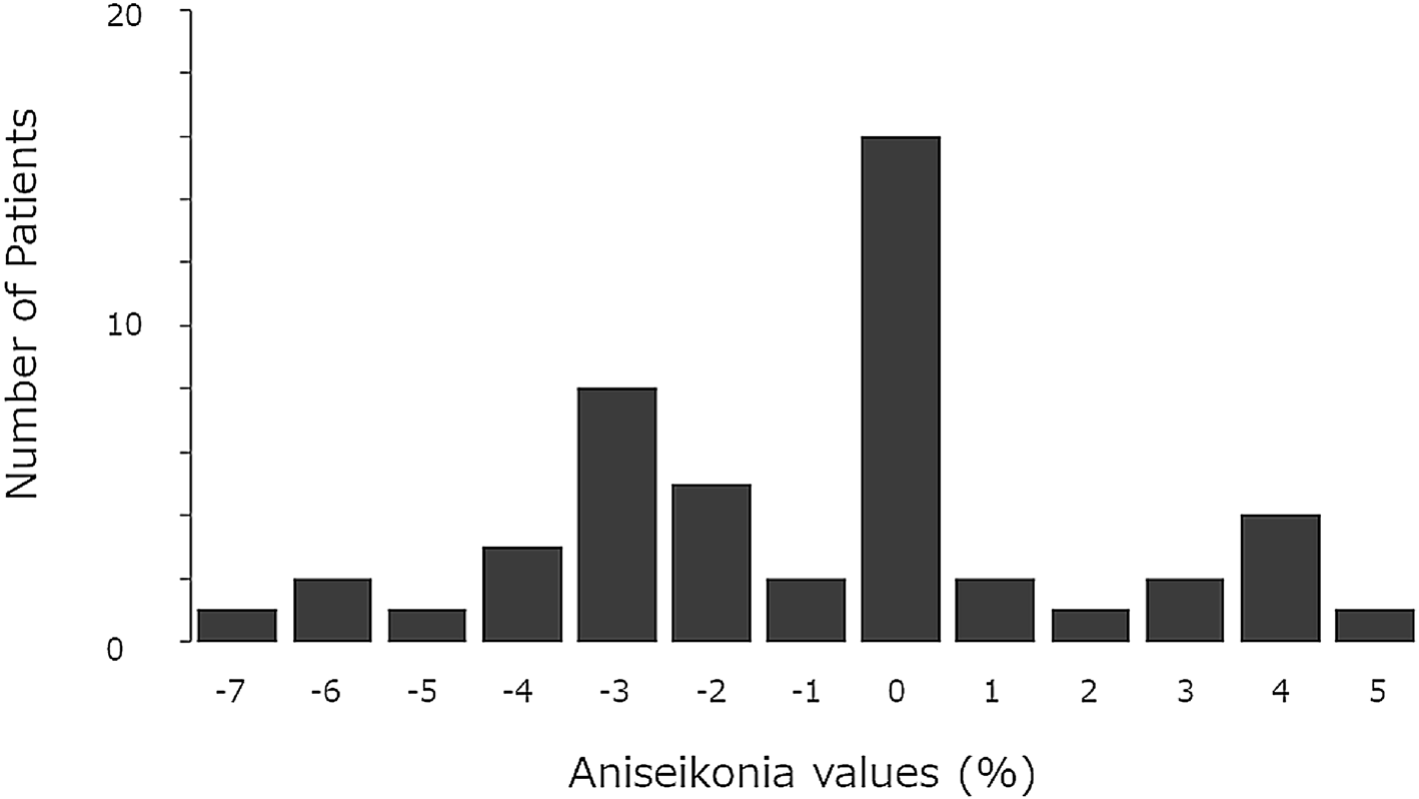
Histogram of the aniseikonia score 12 months after reduced-fluence photodynamic therapy (RFPDT). The value with the highest peak was 0 (n = 16). In this study, 22 (45.8%) and 10 (20.8%) patients had micropsia (defined as aniseikonia value < 0) and macropsia (defined as aniseikonia value > 0), respectively.

This study analyzed 48 eyes of 48 patients. The clinical characteristics of the patients are shown in Table 1. The mean age was 49.2 ± 9.9 years, and the proportion of male patients was 38/48 (79.2%). Two independent raters measured the SRD area. The ICC between the two raters was 0.997. The logMAR BCVA improved significantly 6 (− 0.111 ± 0.148) and 12 (− 0.112 ± 0.200) months after RFPDT compared with that at baseline (− 0.035 ± 0.135) (both *P* < 0.01, with Bonferroni correction) (Fig. 1). The aniseikonia score also improved significantly 6 (2.2 ± 2.3) and 12 (2.2 ± 2.0) months after RFPDT compared with that at baseline (4.1 ± 2.9) (*P* < 0.05 and *P* < 0.01, respectively, with Bonferroni correction) (Fig. 2). All eyes exhibited complete SRD resolution 6 and 12 months after RFPDT. The outer nuclear layer (ONL) thickness at 12 months improved significantly compared with that at baseline (70.3 ± 21.3 vs. 60.8 ± 16.7 μm, *P* < 0.01, data not shown). Of the 48 eyes, 11 showed pigment epithelium detachment (PED) at baseline, and none of the eyes had PED 6 and 12 months after RFPDT. The distribution of the aniseikonia score at 12 months is shown in Fig. 3. Sixteen (33.3%) patients did not have aniseikonia 12 months after RFPDT, whereas 22 (45.8%) patients had micropsia and 10 (20.8%) had macropsia on their affected eyes.

**Table 1:** Clinical characteristics.

Case number, n	48
Age, years	49.2 ± 9.9
Sex (male/female)	38/10
Duration of symptoms, months	9.7 ± 16.3
BCVA at baseline, logMAR	− 0.035 ± 0.135
PDT spot size, μm	3766.0 ± 774.5
ONL thickness at baseline, μm	60.8 ± 16.7
Height of SRD at baseline, μm	200.3 ± 116.8
Width of SRD at baseline, μm	2902.1 ± 1276.0
Area of SRD at baseline, mm^2^	11.1 ± 9.0

BCVA, best-corrected visual acuity; logMAR, logarithm of the minimum angle of resolution; ONL, outer nuclear layer; PDT, photodynamic therapy; SRD, serous retinal detachment.

The correlation between the aniseikonia score at baseline or 12 months and clinical parameters was evaluated. Our results demonstrated that the baseline aniseikonia score significantly correlated with SRD width and area (*P* = 0.003 and *P* = 0.002, respectively) (Table 2). In addition, the aniseikonia score at baseline (*P* = 0.047), ONL thickness at baseline (*P* = 0.027) and 12 months (*P* = 0.014) after RFPDT, baseline SRD area (*P* = 0.005), and EZ disruption (*P* = 0.021) significantly correlated with the aniseikonia score at 12 months (Table 3). There was no significant correlation between the change in aniseikonia score before and 12 months after RFPDT and the time to resolution of SRF after RFPDT (R = 0.078, *P* > 0.05, data not shown). A multiple regression analysis was performed to explore the factors associated with the aniseikonia score 12 months after RFPDT. The results demonstrated that a greater SRD area before PDT in patients with CSC was significantly associated with a higher aniseikonia score 12 months after RFPDT (*P* = 0.034) (Table 4).

**Table 2:** Correlation between the aniseikonia score before reduced-fluence photodynamic therapy and clinical parameters.

	R	*P* value*
Age, years	− 0.100	0.572
Duration of symptoms, months	0.066	0.709
BCVA at baseline, logMAR	0.294	0.092
ONL thickness at baseline, μm	− 0.217	0.217
SRD height at baseline, μm	0.040	0.822
SRD width at baseline, μm	0.497	0.003
SRD area before PDT, mm^2^	0.522	0.002

BCVA, best-corrected visual acuity; logMAR, logarithm of the minimum angle of resolution; ONL, outer nuclear layer; PDT, photodynamic therapy; SRD, serous retinal detachment.

*Spearman’s rank correlation.

**Table 3:** Correlation between the aniseikonia score 12 months after reduced-fluence photodynamic therapy and clinical parameters.

	R	P value*
Age, years	− 0.048	0.744
Duration of symptoms, months	0.177	0.228
Aniseikonia score at baseline	0.344	0.047
BCVA at baseline, logMAR	0.171	0.245
BCVA at 12 months, logMAR	− 0.093	0.530
PDT spot size, μm	0.031	0.835
ONL thickness at baseline, μm	− 0.320	0.027
ONL thickness at 12 months, μm	− 0.353	0.014
SRD height at baseline, μm	− 0.177	0.229
SRD width at baseline, μm	0.248	0.089
SRD area before PDT, mm^2^	0.397	0.005
EZ disruption at 12 months (+/−)	0.331	0.021

BCVA, best-corrected visual acuity; EZ, ellipsoid zone; logMAR, logarithm of the minimum angle of resolution; ONL, outer nuclear layer; PDT, photodynamic therapy; SRD, serous retinal detachment.

*Spearman’s rank correlation.

**Table 4:** Clinical factors related to the aniseikonia score 12 months after reduced-fluence photodynamic therapy.

	β	Standard error	P value*
Aniseikonia score at baseline	− 0.056	0.133	0.773
ONL thickness at baseline, μm	0.124	0.036	0.676
ONL thickness at 12 months, μm	− 0.367	0.026	0.199
SRD area before PDT, mm^2^	0.426	0.040	0.034
EZ disruption at 12 months	0.287	0.709	0.093

EZ, ellipsoid zone; ONL, outer nuclear layer; PDT, photodynamic therapy; SRD, serous retinal detachment.

*Multiple regression analysis.

## Discussion

This study demonstrated that the baseline SRD area was associated with the aniseikonia score 12 months after RFPDT in patients with CSC. To the best of our knowledge, this is the first study regarding the clinical course of aniseikonia and the association between clinical parameters and the aniseikonia score in patients with resolved CSC.

A change in the retinal receptor distribution (stretching or compression) is believed to cause aniseikonia associated with retinal diseases^13^. If the photoreceptors are stretched apart, the image stimulates fewer receptors and is perceived as smaller (micropsia). By contrast, if the photoreceptors are compressed, the image stimulates more receptors and is perceived as larger (macropsia). It is hypothesized that the retina of eyes with CSC is stretched when it is detached^21^. Considering that the baseline SRD width and area were also significantly associated with the baseline aniseikonia score, extensive retinal detachment may lead to retinal stretching and spatial distribution of photoreceptors, resulting in the perception of micropsia in patients with CSC. This abnormal spatial distribution of photoreceptors resulting from the reattached retina appears to be an essential contributing factor to aniseikonia in patients with resolved CSC. In this study, the aniseikonia score was significantly improved 6 and 12 months after RFPDT compared with the baseline score. This may be because foveal structures were restored over time after SRD resolution in patients with CSC^22^.

Our multivariate analysis demonstrated that the SRD area before RFPDT was associated with the aniseikonia score 12 months after treatment. Similarly, Okamoto et al.^23^ examined the relationship between aniseikonia and foveal microstructures after surgery in patients with RRD and found that the aniseikonia value was associated with the retinal detachment area. Shiragami et al.^24^ reported that the extent of retinal detachment and macular status (on/off) were significantly associated with postoperative retinal displacement. They suggested that greater retinal or macular detachment area can cause retinal translocation even after successful surgery. The larger the SRD area, the greater the displacement of photoreceptors, leading to higher aniseikonia in patients with CSC.

In this study, all patients were treated with RFPDT instead of standard PDT. Therefore, the values and changes in aniseikonia scores would have been different if the patients were treated with standard PDT. To date, no studies have reported aniseikonia scores in patients treated with standard PDT. In previous reports comparing standard PDT with RFPDT^25,26^, significant differences were noted in choroidal perfusion and choroidal thickness after PDT, but not in resolution of SRF or visual acuity. In the current study using RFPDT, multivariate analysis showed that the aniseikonia score at 12 months after RFPDT was significantly associated with the baseline SRD area but not with other OCT parameters. Moreover, no significant association was observed between the time to resolution of SRF and the change in aniseikonia score before and after RFPDT. In the future, it would be interesting to investigate whether aniseikonia scores differ between patients treated with standard PDT and those treated with RFPDT.

Consistent with a previous report^10^, visual acuity was significantly improved, and the ONL thickness was significantly increased after treatment. Okamoto et al.^23^ demonstrated a significant association between postoperative visual acuity and aniseikonia value in patients with RRD after surgery. In the present study, the ONL thickness at baseline and 12 months, not visual acuity, significantly correlated with the aniseikonia score (*P* = 0.027 and 0.014, respectively) in the univariate analysis. Matsumoto et al.^27^ previously demonstrated that visual outcomes correlated with ONL thickness in eyes with resolved CSC. Since both BCVA at baseline and 12 months were good (− 0.035 ± 0.135 and − 0.112 ± 0.200, respectively), the ONL thickness, not BCVA showed a significant correlation with the aniseikonia score in our study.

Previously^10^, we showed that the degree of metamorphopsia was associated with baseline ONL thickness in patients with resolved CSC with good baseline BCVA after RFPDT. Therefore, early treatment before the decrease in ONL thickness might prevent metamorphopsia. Conversely, the aniseikonia score was associated only with the area of the SRD. Aniseikonia may occur once the retina is widely detached in patients with CSC, similar to that in patients with RRD. Here, early treatment to prevent aniseikonia is not necessary. However, treatment may help reduce the sequelae when the SRD area tends to enlarge. Further research is needed to explore whether aniseikonia occurs when the retina is widely detached even once or when the retina is widely detached for a considerable period.

This study has some limitations. First, the study has a relatively small sample size. Second, we did not investigate micropsia and macropsia separately, although a difference was possible in the mechanism that causes micropsia and macropsia. Third, the SRD area is not accurate in some patients because SRD spontaneously disappears and develops in the eyes with CSC. Therefore, further studies with larger sample sizes are needed.

In conclusion, aniseikonia resolved after RFPDT; however, nearly two-thirds of the patients had aniseikonia 12 months after RFPDT. Patients with CSC with a larger SRD area may have aniseikonia after SRD resolution.

## Data Availability

The datasets used and/or analyzed during the current study available from the corresponding author on reasonable request.
